# Time spent at health facility is a key driver of patient satisfaction, but did not influence retention to HIV care: A serial cross-sectional study in Mozambique

**DOI:** 10.1371/journal.pone.0299282

**Published:** 2024-04-18

**Authors:** Caroline De Schacht, Gustavo Amorim, Lázaro Calvo, Efthymios Ntasis, Sara Van Rompaey, Julieta Matsimbe, Samuel Martinho, Erin Graves, Maria Fernanda Sardella Alvim, Ann Green, Hidayat Kassim, Inoque Carlos Carlos, C. William Wester, Carolyn M. Audet

**Affiliations:** 1 Friends in Global Health (FGH), Maputo, Mozambique; 2 Department of Biostatistics, Vanderbilt University Medical Center (VUMC), Nashville, Tennessee, United States of America; 3 Friends in Global Health (FGH), Quelimane, Mozambique; 4 Vanderbilt Institute for Global Health (VIGH), Vanderbilt University Medical Center (VUMC), Nashville, Tennessee, United States of America; 5 Provincial Health Directorate of Zambézia, Quelimane, Mozambique; 6 United Nations Funds for Development (UNFDP), Beira, Mozambique; 7 Operations Research Unit, Provincial Health Directorate of Zambézia, Quelimane, Mozambique; 8 Department of Medicine, Division of Infectious Diseases, Vanderbilt University Medical Center (VUMC), Nashville, Tennessee, United States of America; 9 Department of Health Policy, Vanderbilt University Medical Center (VUMC), Nashville, Tennessee, United States of America; Clinton Health Access Initiative, UNITED STATES

## Abstract

**Introduction:**

Patient satisfaction with clinical services can have an effect on retention in HIV care and adherence to antiretroviral therapy. This study assessed patient satisfaction and its association with retention and viral suppression in Zambézia Province, Mozambique.

**Methods:**

Monthly exit interviews with persons living with HIV were completed from August 2017-January 2019 in 20 health facilities; clinical data were extracted from medical records. Regression analyses assessed the effect of satisfaction scores on retention and viral suppression, adjusting for age, sex, education, civil status, time on treatment, and site. Satisfaction scores were correlated with time spent at health facilities using generalized linear regression models.

**Results:**

Data from 4388 patients were analyzed. Overall median satisfaction score was 75% (IQR 53%-84%); median time spent at facilities (from arrival until completion of clinical services) was 2h54min (IQR 1h48min-4h). Overall satisfaction score was not associated with higher odds of retention or viral suppression, but association was seen between satisfaction regarding attention given to patient and respect and higher odds of viral suppression. Patient satisfaction was negatively associated with time spent in facility (Spearman’s correlation -0.63). Increased time spent at facility (from 1 to 3 hours) was not associated with lower retention in care (OR 0.72 [95%CI:0.52–1.01] and 0.83 [95%CI: 0.63–1.09] at 6- and 12-months, respectively), nor with a lower odds of viral suppression (OR 0.96 [95%CI: 0.71–1.32]).

**Conclusions:**

Strategies to reduce patient wait times at the health facility warrant continued prioritization. Differentiated models of care have helped considerably, but novel approaches are still needed to further decongest crowded health facilities. In addition, a good client-provider communication and positive attitude can improve patient satisfaction with health services, with an overall improved retention.

## Introduction

Approximately 58% of all new HIV cases occurred in the sub-Saharan African (SSA) region in 2021 [1]. Mozambique continues to rank among the ten countries most severely affected by HIV, with 2,097,000 adult Mozambicans living with HIV in 2020. Combination antiretroviral therapy (ART) has been available in Mozambique since 2006, with 1,698,486 persons living with HIV (PLWH) currently receiving ART by the end of 2021 [2], with recent data highlighting that 94.6% of adults living with HIV aware of their status on ART (97.5% for females and lower for males at 94.3%). The success of the country’s HIV program hinges on patient retention in longitudinal HIV care and adherence to prescribed ART.

Health system responsiveness (HSR) is based on patients’ experiences within the health system and focuses on eight domains: autonomy, support systems, confidentiality, quality of basic amenities, communication, access, promptness, and dignity [3]. Patient satisfaction, a construct related to several HSR domains, is a more subjective measure of a patient’s perceived needs and expectations for health system interactions and has been described elsewhere [4–6]. Efforts to improve understanding of patient satisfaction are important not only for use as an independent indicator of health system effectiveness, but also for its strong association with increased retention in care [7, 8]. However, associations between HSR, patient satisfaction, and retention in HIV services have not been well documented in resource-constrained settings, especially rural areas of Mozambique.

With funding from the United States President’s Emergency Plan for AIDS Relief through the United States Centers for Disease Control and Prevention (CDC), Friends in Global Health (FGH), a subsidiary of Vanderbilt University Medical Center (VUMC), has been providing technical assistance for HIV care and treatment services in Zambézia Province since 2006, currently providing support to 149 health facilities (HF).

This study, led by FGH/VUMC and provincial health authorities, aimed to better understand patients’ level of satisfaction with their received care at 20 supported health facilities and perceptions of health system responsiveness–particularly the domains of confidentiality, communication, dignity, promptness–and the impact on retention in HIV care and viral suppression.

## Methods

### Study setting

**Fig 1:**
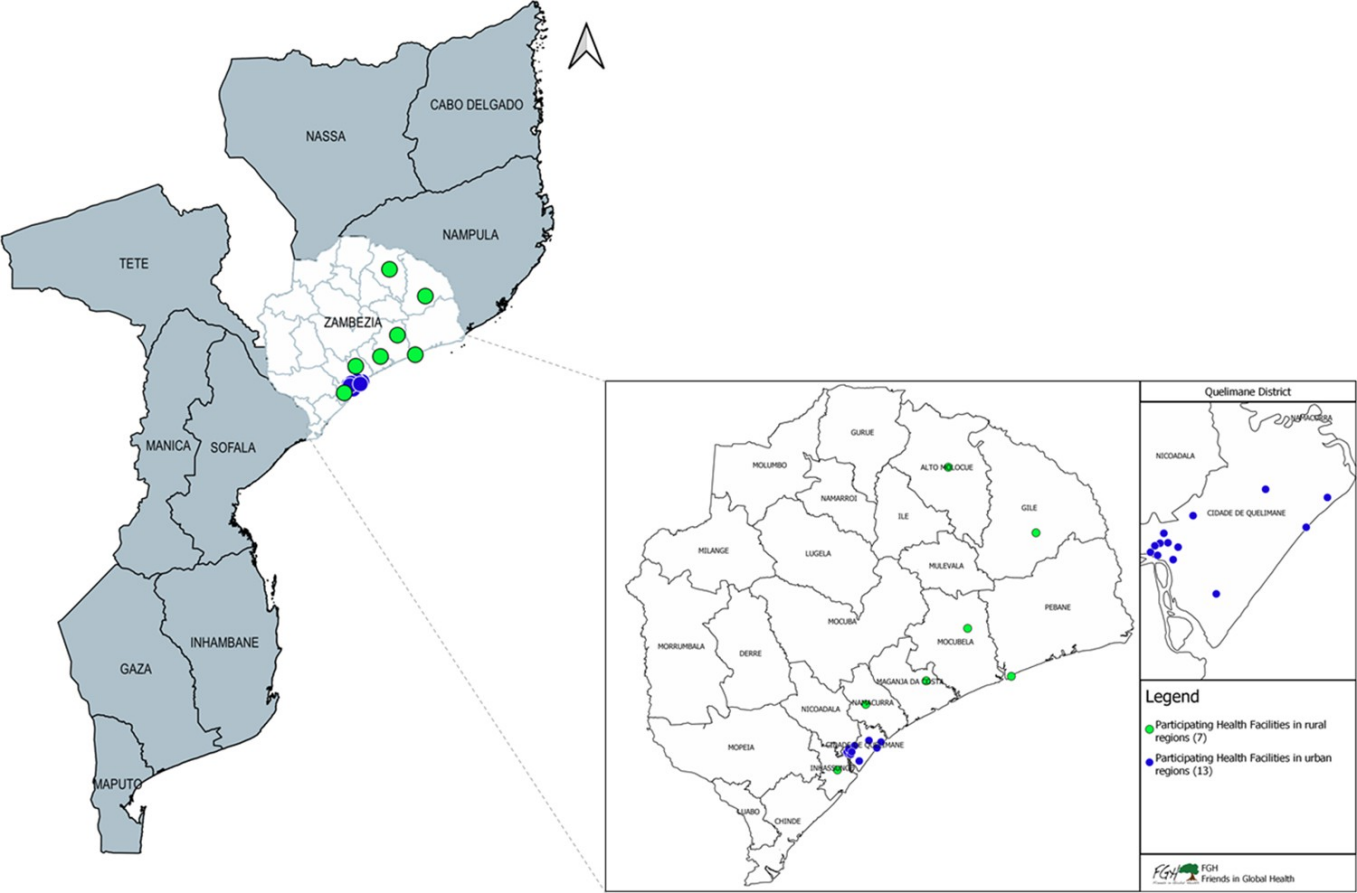
Study locations (n = 20) (green = rural; dark blue = urban).

The study was conducted in Zambézia, a province located in central Mozambique, with a population of 5.5 million people (about 19% of the total population) [9]. In 2021, the HIV prevalence was estimated at 12.5% (among persons 15+ years of age) [10, 11]. Program data showed a 12-month retention to HIV services during the study period ranging from 68% and 71% [12, 13]. Our study was implemented in 20 HF in total, specifically, the main HF located within 7 rural districts (Alto Molócuè, Gilé, Inhassunge, Maganja da Costa, Mocubela, Namacurra, and Pebane) and 13 HF located in the more urban provincial capital district, Quelimane City (**Fig 1 [14]**). Two (10%) of the 20 HF were larger referral-level hospitals that also offer primary health care (“secondary HF”), while the remaining 18 (90%) HF were smaller primary health care facilities (“primary HF”), that ranged in size based on population within the respective district. No other HF characteristics were captured.

### Design

Monthly interviewer-assisted exit surveys were employed to collect patient satisfaction indicators among consenting persons living with HIV between August 2017 and February 2019. The satisfaction survey was based on other patient satisfaction questionnaires (CSQ-8, PSQ-18) [15, 16], and adapted to local context, removing items related to factors like health insurance and direct payments as not relevant to the country’s context. In addition, a chart review for all consenting participants was done to assess retention in care and adherence outcomes after the survey, using data up to 24 months after enrolment for the survey (August 2017-February 2021). Eligibility criteria for participants included being 18 years of age or older and being enrolled in HIV care and treatment at one of the 20 participating health facilities.

### Study procedures

A convenience sampling strategy was used to recruit participants. Potential participants who attended the adult ART clinic, Maternal Child Health (MCH)/ Prevention of Mother-to-Child Transmission (PMTCT) clinic and/or the HIV/tuberculosis (TB) clinic were approached as they left the health facility. A face-to-face interview was conducted after explaining procedures, confirming eligibility, and obtaining informed consent from each participant. A structured survey instrument was used, with a 4-tiered Likert-scale response option available, covering eight components of patient satisfaction (*i*.*e*., wait time [time from arrival until being attended]; opportunity to discuss health needs and concerns; respect; feeling listened to by health staff; staff’s explanation of health issues; opportunity to ask questions to health staff; helpfulness of health staff; and general satisfaction), and questions regarding time a person spent travelling to and staying at the health facility. Data were recorded using mobile cellular devices that were stored in a secure web-based application for building survey databases (REDCap™ electronic data capture tools, hosted at Vanderbilt University Medical Center) [17, 18]. The interview was done in the participant’s preferred language (e.g., Portuguese, Chuabo, Lomué, Munhawa, Makua). Clinical data regarding routinely collected HIV data such as World Health Organization (WHO) clinical staging, ART refills, CD4 cell count, and viral load results were extracted from electronic patient medical records available at the health facility. Data collected were de-identified for analysis purposes.

### Statistical aspects

We powered our evaluation sample size to detect a 2-point change in patient satisfaction score, assuming a standard deviation [sd] of 1.8 in the satisfaction scale, 80% power and a type 1 error set to 5%. We estimated that a total sample size of 6,048 for the intended 24 health facilities providing data over 18 months, or 14 patients per facility per month, would be sufficient. Original power estimates were based on 24 HF that were planned to provide data. However, our final sample size was smaller as four districts were no longer supported by FGH/VUMC at the start of the data collection, so it was not possible to collect data from these sites. Repeated interviews were excluded from all analyses to avoid potential bias, whereby if more than one interview per patient was completed, only the last interview was included in the final analysis.

Continuous variables were presented as medians (with interquartile ranges [IQR]) and frequency tables (percentages) were used to summarize categorical variables. Satisfaction was scored using a 4-tiered Likert scale, varying from 1 to 4 (for each of the eight components), leading to a maximum score of 32. Spearman’s correlation was used to assess the correlation between the combined satisfaction scores and self-reported time spent in the HF. Univariable generalized linear mixed effects models with a random intercept were used to evaluate the association of baseline variables and main outcomes: 6-month retention, 12-month retention, and viral suppression. Health facilities were used as clusters to account for possible correlations within patients from the same site. Multivariable generalized linear mixed effects models we used to assess the effect of combined patient satisfaction scores on 6- and 12-month retention, as well as on viral load, using restricted cubic splines to alleviate linearity assumptions. We used three knots placed at the 5^th^, 50^th^, and 95^th^ percentiles. Interpretation of non-linear variables was done via contrasts [19]. Impact of non-linear variables in the final model was assessed via likelihood-ratio tests. All regressions were adjusted for age, sex, education, civil status, years on ART, and location/type of HF (*i*.*e*., rural or urban). Individual logistic regressions with mixed effects, again with health facilities as clusters, measured the impact of individual satisfaction questions on retention and viral load, adjusting for the same factors. The overall time spent in the health facility was computed for each patient and its impact on retention and viral load was also computed [20]. Time spent in the health facility was modelled via restricted cubic splines, adjusting for the same covariates as above: age at interview date, location (urban/rural), sex, education, marital status. We used again three knots placed at the 5^th^, 50^th^, and 95^th^ percentiles. We later discretized the time spent in health facility into three groups, short (< 2.5 hours), medium (between 2.5 and 5 hours), and long (> 5 hours), and its impact on the combined score was assessed via ordinal regression. Multiple imputation with chained equations (MICE) were used to impute missing information on all variables with missing information (marital status, education level, and time on ART) prior to all multivariable regressions. We used 20 imputations, with 50 iterations; and convergence plots (mean and standard deviations) were used for diagnostics (**S1 Fig**). Statistical analyses were conducted for each imputed dataset and final estimates were combined using Rubin’s rules [21]. As the majority of missingness comes from a single variable (marital status), we performed sensitivity analysis excluding this variable from all multivariable models. A value of p<0.05 was considered statistically significant. The R statistical software [R Core Team (2018)] supported the quantitative analysis [22].

### Definitions

For the purposes of this evaluation and results interpretation, we defined patient satisfaction as a patient’s perception that their expectations were met for health care-related services they received and interactions with providers, where eight components were considered as described above. Time at health facility was defined as the self-reported length of time from when the patient arrived at the HF to the time they exited the health facility complex. Retention outcomes were defined as completing a scheduled clinic visit or an ART pick-up that was within the time interval of 59 days to end of the retention period of interest (*i*.*e*., 6- or 12-months post-survey). Viral suppression was defined as having an HIV viral load of < 1,000 copies/ml.

### Ethical aspects

The protocol and instruments were approved by the provincial-level local ethics committee (Institutional Health Ethics Committee of Zambézia [Reference 03/CIBS-Z/16] and the VUMC Institutional Review Board [#160777]). The protocol was reviewed in accordance with the CDC human research protection procedures and was determined to be research, with CDC investigators not being formally engaged in the research activities. All participants provided written informed consent prior to data collection.

## Results

### Sociodemographic characteristics

A total of 4,388 participants responded to the survey, with a total of 5,040 interviews done. Refusal rate was reported to be low, at approximately 5%. For patients with more than one completed survey, only the last was included in the analysis. Of the study population, median age was 30 years (interquartile range [IQR] 25–38), 3,263 (74%) were female and 3,248 (74%) had no formal or primary education. Very few (n = 66, 1.5%) participants reported that their native language was Portuguese; half (n = 2170) said they were attended to at the HF in their preferred language. Marital status was missing for 938 (28.3%) participants, while education levels and time on ART were missing for 16 (<1%) and 120 (3.6%) participants, respectively. During the HF visit (preceding the interview), 1,017 (23%) participants reported receiving more than three services (**Table 1**). Among the 4,388 interviewees, 3,318 (76%) had clinical data available in the electronic patient database. Characteristics of interviewees having data available within the electronic patient database were, overall, similar to the total study population, with a median age at interview of 30 years (IQR 25–38 years); 2,459 (74%) were female and 2,451 (74%) had no formal or had a primary level education. Approximately two-thirds (2,156; 65%) were patients attended in urban HF. The median time on ART was equal to 1.5 years (IQR 0.5–3.7) (**Table 1**).

**Table 1:** Sociodemographic characteristics of the study population (n = 4,388) and study population with clinical data available (n = 3,318).

	Survey study population (n, %)	Study population with clinical data (n, %)
	(n = 4,388)	(n = 3,318)
Age at time of interview, years (median, IQR)	30.0 [25.0;38.0]	30.0 [25.0;38.0]
Sex		
Female	3,263 (74.4%)	2,459 (74.1%)
Male	1,125 (25.6%)	859 (25.9%)
District		
Alto Molócuè	219 (5.0%)	168 (5.1%)
Quelimane	2,873 (65.5%)	2,156 (65.0%)
Gilé	220 (5.0%)	166 (5.0%)
Inhassunge	210 (4.8%)	155 (4.7%)
Maganja da Costa	218 (5.0%)	166 (5.0%)
Mocubela	212 (4.8%)	164 (4.9%)
Namacurra	214 (4.9%)	169 (5.1%)
Pebane	222 (5.1%)	174 (5.2%)
Urban/ Rural		
Urban	2,873 (65.5%)	2,156 (65.0%)
Rural	1,515 (34.5%)	1,162 (35.0%)
Level of education		
No education	949 (21.6%)	710 (21.4%)
Primary education	2,299 (52.4%)	1,741 (52.5%)
Secondary education	1,049 (23.9%)	794 (23.9%)
Technical School	21 (0.5%)	14 (0.4%)
University	52 (0.5%)	43 (1.3%)
No information	18 (0.4%)	16 (0.5%)
Marital status		
Living with partner	NA	1147 (34.6%)
Married	NA	452 (13.6%)
Single	NA	629 (19.0%)
Widowed	NA	152 (4.6%)
Separated	NA	2 (0.1%)
No information	NA	936 (28.2%)
Native language		
Chuabo	2583 (58.9%)	1938 (58.4%)
Elomwé	605 (13.8%)	443 (13.4%)
Muniga	525 (12.0%)	417 (12.6%)
Nharinga	251 (5.7%)	201 (6.1%)
Portuguese	66 (1.5%)	56 (1.7%)
Other	341 (7.8%)	260 (7.8%)
No response	17 (0.4%)	3 (<0.1%)
Self-reported comprehension of spoken Portuguese		
Fluent	2,107 (48.0%)	1,689 (50.9%)
A little	1,979 (45.1%)	1,414 (42.6%)
Not	302 (6.9%)	215 (6.5%)
Attendance at HF in the preferred language?		
Yes	2,170 (49.5%)	1,675 (50.5%)
No	2,215 (50.5%)	1,640 (49.4%)
No response	3 (<0.1%)	3 (0.06%)
Median time spent at the HF, hours (median, IQR)	2h55m (1h46m-4h10m)	2h55m (1h41m-4h15m)
Median satisfaction score (IQR)	24.0 [17.0;27.0]	24.0 [16.0;26.0]
Reason for HF visit (*check all that apply*)		
HIV testing	210 (4.8%)	168 (5.1%)
ART care	246 (5.6%)	188 (5.7%)
Routine HIV care	4,041 (92.1%)	3058 (92.2%)
Non-routine HIV care	106 (2.4%)	80 (2.4%)
Other non-HIV care	197 (4.5%)	165 (5.0%)
Services received at HF (*check all that apply*)		
Adult ART Care	3,217 (73.3%)	2,430 (73.2%)
Pediatric ART Care	382 (8.7%)	280 (8.4%)
TB-HIV coinfection	398 (9.1%)	285 (8.6%)
Youth Clinic	39 (0.9%)	32 (1.0%)
Antenatal Care Clinic	651 (14.8%)	511 (15.4%)
Child-at-risk Clinic	780 (17.8%)	556 (16.8%)
Laboratory	894 (20.4%)	699 (21.1%)
Pharmacy	3,619 (82.5%)	2,770 (83.5%)
Psychosocial support	2,087 (47.6%)	1,656 (49.9%)
Other	539 (12.3%)	402 (12.1%)
Number of services received at HF		
0	2 (<0.1%)	1 (<0.1%)
1	91 (2.1%)	47 (1.4%)
2	1,558 (35.5%)	1,129 (34.0%)
3	1,720 (39.2%)	1,369 (41.3%)
4	841 (19.2%)	646 (19.5%)
5	170 (3.9%)	122 (3.7%)
6	6 (<0.1%)	4 (0.1%)
CD4 cell count at HIV enrolment	NA	511 [30;705]
Time since ART initiation (years)	NA	1.53 [0.53;3.69]
Pregnancy status at enrollment (women only)		
Yes	NA	1,104 (51.%)
No	NA	1,186 (48.2%)
CAG at enrollment		
Yes	NA	423 (12.7%)
No	NA	2,895 (87.3%)

Abbreviations NA: Not available; CAG: Community Adherence Group; IQR: interquartile range; HF: Health Facility

### Patient satisfaction scores (all surveyed participants)

**Fig 2:**
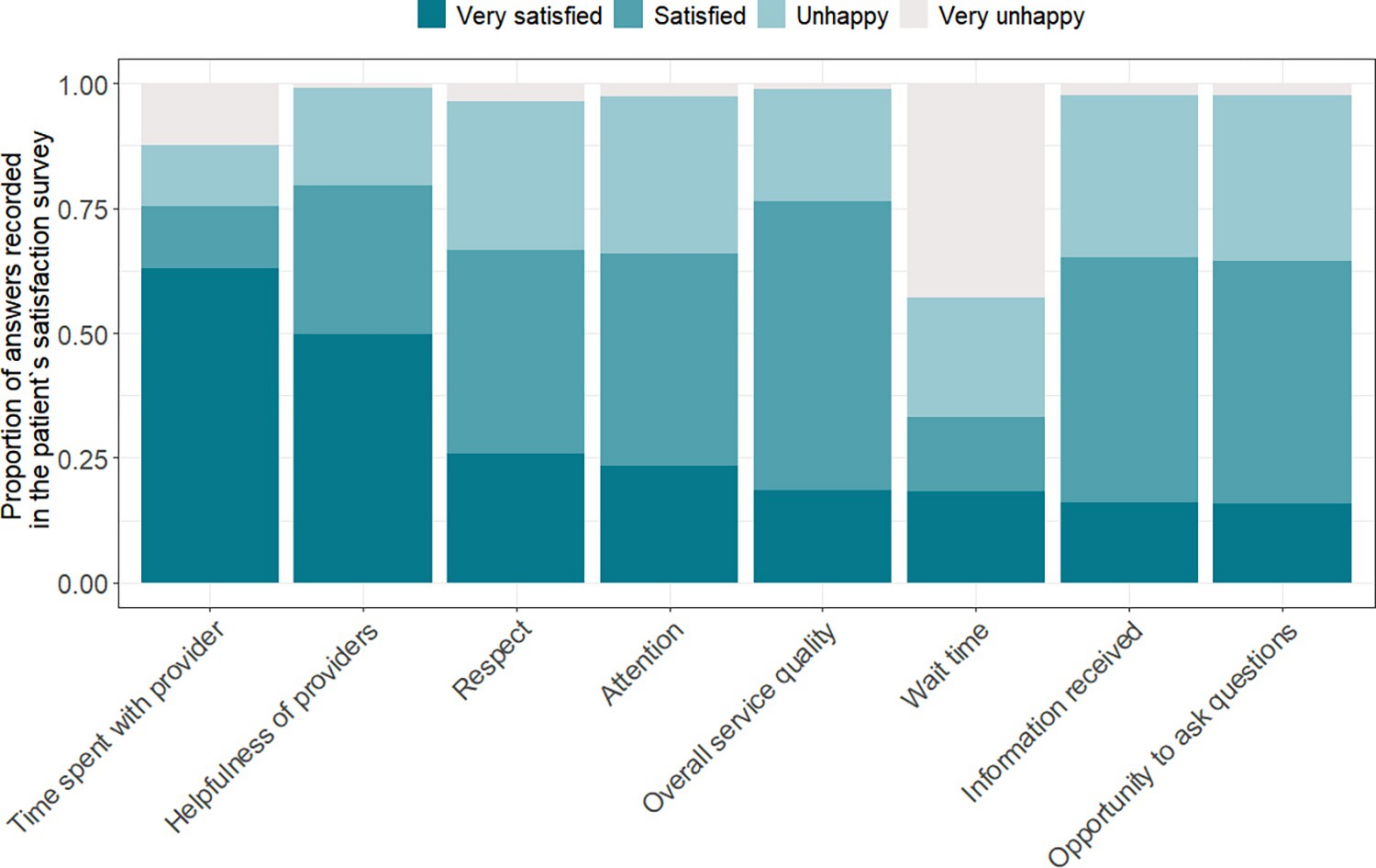
Satisfaction score per individual question (n = 4,388).

The overall median patient satisfaction score was 24 (out of a possible total score of 32) or 75% (IQR 53%-84%). Each of the individual components, namely, time spent with provider, helpfulness, and overall service were considered good or very good (scores 3 or 4, respectively) for more than 75% of the patients throughout the study period. Conversely, more than 66% of the patients were unhappy or very unhappy (scores 2 and 1, respectively) with waiting times (**Fig 2**).

**Fig 3:**
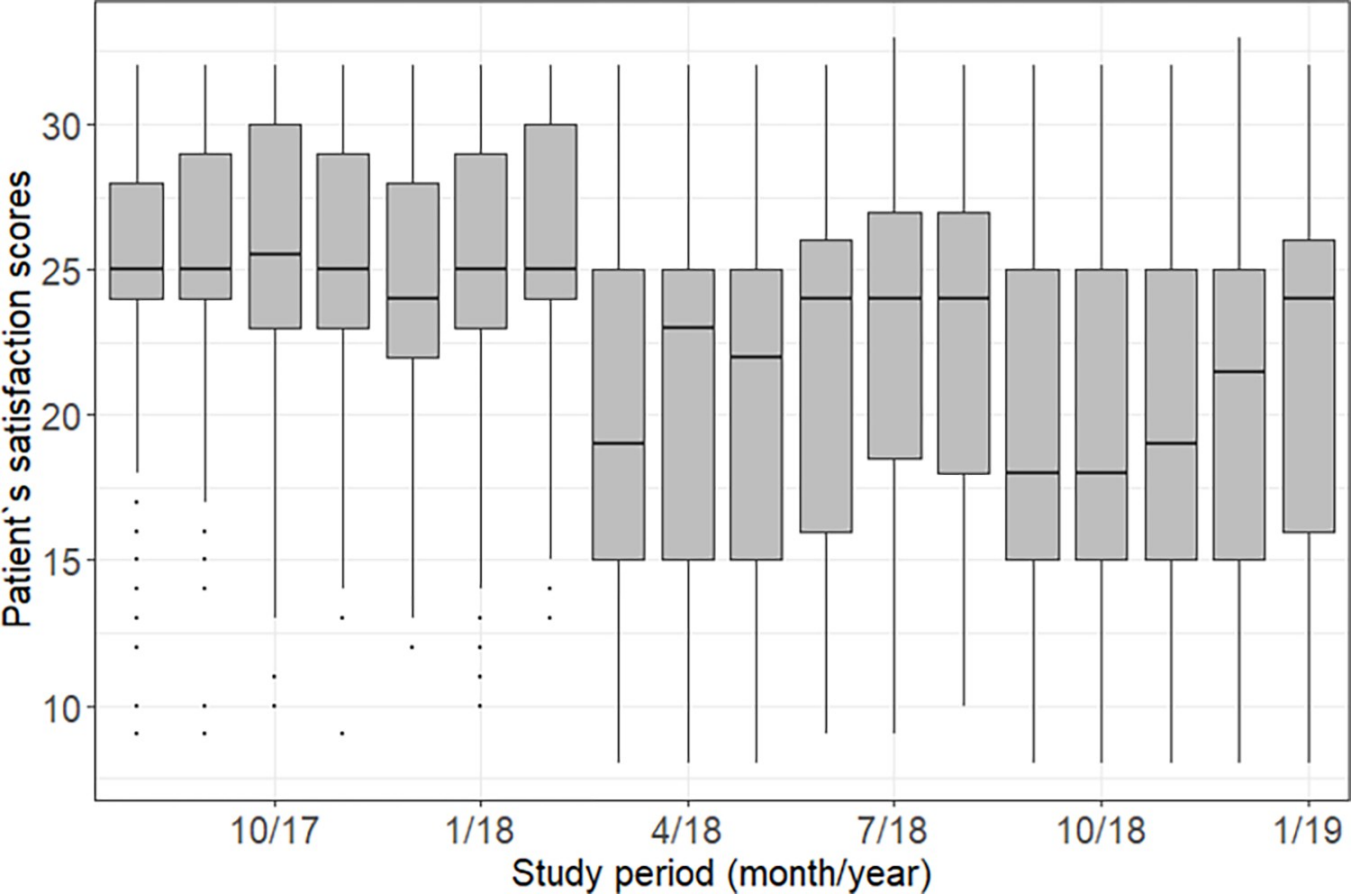
Boxplots by study period, showing the variation of patient satisfaction scores over time.

We observed a variation between districts, with the lowest overall median satisfaction in Gilé and Pebane (59%, IQR 47%-78%) and the highest in Quelimane district 78% (69%-91%) (**S2 Fig**). Patients receiving care at health facilities located in urban settings, on average, had higher scores than patients receiving care at rural HF (**Table 2**). There was a decrease in satisfaction scores observed in almost all districts after February 2018, as shown by the boxplot in **Fig 3**: the median values decreased from 25 (IQR 24–30) from February 2018 to 19 (IQR 15–25) in March 2018, but then increased again to 24 (IQR16-26) in January 2019.

**Table 2:** Patient characteristics, per satisfaction score category (n = 4,384).

	Total (n = 4,384†)	Satisfaction Score <24 (n = 1,815)	Satisfaction Score > = 24 (n = 2,569)
Age, years (median, IQR)	30.0 [25.0;38.0]	30.0 [24.0;37.0]	30.0 [25.0;38.0]
Sex			
Female	3,260 (74.4%)	1,383 (76.2%)	1,877 (73.1%)
Male	1,124 (25.6%)	432 (23.8%)	692 (26.9%)
District			
Alto Molócuè	219 (5.0%)	137 (7.5%)	82 (3.2%)
Quelimane	2,870 (65.5%)	901 (49.6%)	1,969 (76.6%)
Gilé	220 (5.0%)	141 (7.8%)	79 (3.1%)
Inhassunge	210 (4.8%)	119 (6.6%)	91 (3.5%)
Maganja da Costa	217 (4.9%)	117 (6.4%)	100 (3.9%)
Mocubela	212 (4.8%)	140 (7.7%)	72 (2.8%)
Namacurra	214 (4.9%)	114 (6.3%)	100 (3.9%)
Pebane	222 (5.1%)	146 (8.0%)	76 (3.0%)
Rural/ Urban			
Rural	1,514 (34.5%)	914 (50.4%)	600 (23.4%)
Urban	2,870 (65.5%)	901 (49.6%)	1,969 (76.6%)
Level of education			
No education	947 (21.6%)	432 (23.8%)	515 (20.0%)
Primary education	2,297 (52.4%)	959 (52.8%)	1,338 (52.1%)
Secondary education	1049 (23.9%)	400 (22.0%)	649 (25.3%)
Technical	21 (0.5%)	5 (0.3%)	16 (0.6%)
University	52 (1.2%)	11 (0.6%)	41 (1.6%)
No response	18 (0.4%)	8 (0.4%)	10 (0.4%)
Native language			
Chuabo	2583 (58.9%)	911 (50.2%)	1672 (65.1%)
Elomwé	605 (13.8%)	362 (19.9%)	243 (9.5%)
Muniga	525 (12.0%)	284 (15.6%)	241 (9.4%)
Nharinga	251 (5.7%)	112 (6.2%)	139 (5.4%)
Portuguese	66 (1.5%)	24 (1.3%)	42 (1.6%)
Other	341 (7.8%)	117 (6.4%)	224 (8.7%)
No response	13 (0.3%)	5 (0.3%)	8 (0.3%)
Self-reported comprehension of spoken Portuguese			
Fluent	2,106 (48.0%)	818 (45.1%)	1,288 (50.1%)
A little	1,976 (45.1%)	822 (45.3%)	1,154 (44.9%)
Not	302 (6.9%)	175 (9.6%)	127 (4.9%)
Attendance at HF in the preferred language?			
Yes	2,168 (49.5%)	565 (31.1%)	1,603 (62.4%)
No	2,213 (50.5%)	1,247 (68.7%)	966 (37.6%)
No response	2 (<0.1%)	2 (0.1%)	0 (0.0%)
Median time spent at the HF, hours (median, IQR)	2h55m (1h46m-4h10m)	4h02m (3h09m-5h00m)	2h05m (1h20m-3h02m)
Reason for HF visit (*check all that apply*)			
HIV testing	210 (4.8%)	78 (4.3%)	132 (5.1%)
ART care	246 (5.6%)	85 (4.7%)	161 (6.3%)
Routine HIV care	4,038 (92.1%)	1,686 (92.9%)	2,352 (91.6%)
Non-routine HIV care	105 (2.4%)	48 (2.6%)	57 (2.2%)
Other non-HIV care	197 (4.5%)	80 (4.4%)	117 (4.6%)
Services received at HF (*check all that apply*)			
Adult ART Care	3,215 (73.3%)	1,176 (64.8%)	2,039 (79.4%)
Pediatric ART Care	382 (8.7%)	185 (10.2%)	197 (7.7%)
TB-HIV coinfection	396 (9.0%)	113 (6.2%)	283 (11.0%)
Youth Clinic	39 (0.9%)	21 (1.2%)	18 (0.70%)
Antenatal Care Clinic	651 (14.8%)	277 (15.3%)	374 (14.6%)
Child-at-risk Clinic	779 (17.8%)	372 (20.5%)	407 (15.8%)
Laboratory	894 (20.4%)	404 (22.3%)	490 (19.1%)
Pharmacy	3,618 (82.5%)	1,601 (88.2%)	2,017 (78.5%)
Psychosocial support	2,086 (47.6%)	921 (50.7%)	1,165 (45.3%)
Other	538 (12.3%)	239 (13.2%)	299 (11.6%)

†Four patients without satisfaction score in at least one of the questions could not be included. Abbreviations: IQR: interquartile range; HF: Health facility

### Satisfaction score and retention in care (population with clinical data available)

The median patient satisfaction score among patients with clinical data available was 75% (IQR 50%-82%). Retention at 6-months and at 12-months post-interview was 91% (3,022/3,318) and 86% (2,864/3,318), respectively (**Table 3**). Women were retained more frequently compared to men at 6-months (92% versus 88%, p<0.01) and 12-months (88% versus 82%, p<0.01). Older patients were more likely to be retained at 6 and 12-months (p<0.01).

**Table 3:** Patient’s characteristics stratified by retention status at 6- months post-exit interview (n = 3318), 12-months post-exit interview (n = 3318), and viral suppression within 1-year post-exit interview (n = 1718).

	6-months retention			12-months retention			Viral suppression		
	Not retained (n, %)	Retained (n, %)	p-value*	Not retained (n, %)	Retained (n, %)	p-value*	Not virally suppressed (n, %)	Virally suppressed (n, %)	p-value*
	(n = 296)	(n = 3,022)		(n = 454)	(n = 2,864)		(n = 421)	(n = 1,297)	
Satisfaction Score	23.0 [17.0;27.0]	24.0 [16.0;26.0]	0.729	24.0 [17.0;26.0]	24.0 [16.0;26.0]	0.901	22.0 [15.0;25.0]	24.0 [16.0;26.0]	**0.049**
Sex			**<0.001**			**<0.001**			**<0.001**
Female	181 (61.1%)	2,180 (72.1%)		285 (62.8%)	2,076 (72.5%)		270 (64.1%)	1,002 (77.3%)	
Male	115 (38.9%)	842 (27.9%)		169 (37.2%)	788 (27.5%)		151 (35.9%)	295 (22.7%)	
Age at baseline	28.0 [23.0;34.0]	30.0 [25.0;38.0]	**<0.001**	29.0 [23.0;35.0]	31.0 [25.0;39.0]	**<0.001**	23.0 [9.00;30.0]	28.0 [22.0;35.0]	**<0.001**
Marital status			**0.044**			0.083			**<0.001**
Living with partner	89 (30.1%)	1,058 (35.0%)		133 (29.3%)	1,014 (35.4%)		117 (27.8%)	488 (37.6%)	
Married	41 (13.9%)	411 (13.6%)		59 (13.0%)	393 (13.7%)		45 (10.7%)	171 (13.2%)	
Single	71 (24.0%)	558 (18.5%)		104 (22.9%)	525 (18.3%)		59 (14.0%)	252 (19.4%)	
Separated	1 (0.3%)	1 (<0.1%)		0 (0.00%)	2 (0.07%)		0 (0%)	0 (0%)	
Widowed	8 (2.7%)	144 (4.8%)		20 (4.41%)	132 (4.61%)		11 (2.61%)	68 (5.24%)	
No response	86 (29.1%)	850 (28.1%)		138 (30.4%)	798 (27.9%)		507 (29.5%)	318 (24.5%)	
Education level			**0.022**			0.122			0.241
Primary or less	202 (68.2%)	2,249 (74.4%)		318 (70.0%)	2,133 (74.5%)		295 (71.1%)	925 (71.6%)	
Secondary/higher	94 (31.8%)	757 (25.0%)		136 (30.0%)	715 (25.0%)		120 (28.9%)	366 (28.4%)	
No response	0 (0.0%)	16 (0.5%)		0 (0.0%)	16 (0.6%)		0 (0%)	0 (0%)	
Rural/ Urban			0.561			0.771			**<0.001**
Rural	98 (33.1%)	1,064 (35.2%)		153 (33.7%)	1,009 (35.2%)		232 (55.1%)	444 (34.2%)	
Urban	198 (66.9%)	1,958 (64.8%)		301 (66.3%)	1,855 (64.8%)		189 (44.9%)	853 (65.8%)	
Enrolled in CAG			**<0.001**			**<0.001**			0.112
No	283 (95.6%)	2,612 (86.4%)		433 (95.4%)	2,462 (86.0%)		371 (88.1%)	1,132 (87.3%)	
Yes	13 (4.4%)	410 (13.6%)		21 (4.63%)	402 (14.0%)		50 (11.9%)	165 (12.7%)	
Pregnancy status at enrollment (women only)			0.518			0.490			0.128
No	91 (50.3%)	1,065 (48.9%)		145 (50.9%)	1,011 (48.7%)		142 (52.6%)	469 (46.8%)	
Yes	78 (43.1%)	1,017 (46.7%)		124 (43.5%)	971 (46.8%)		127 (47.0%)	522 (52.1%)	
No response	12 (6.7%)	98 (4.5%)		16 (5.6%)	94 (4.5%)		1 (0.4%)	11 (1.1%)	
TB/HIV co-infection			**0.001**			**<0.001**			**<0.001**
No	193 (65.2%)	1,715 (56.8%)		290 (63.9%)	1,618 (56.5%)		161 (38.2%)	755 (58.2%)	
Yes	12 (4.1%)	69 (2.3%)		21 (4.63%)	60 (2.09%)		10 (2.38%)	26 (2.00%)	
No information	91 (30.7%)	1,238 (41.0%)		143 (31.5%)	1,186 (41.4%)		250 (59.4%)	516 (39.8%)	
Time in spent in the HF (hours)	3.0 [2.0;4.2]	2.9 [1.7;4.2]	0.276	3.00 [1.83;4.33]	2.87 [1.67;4.22]	0.072	3.20 [2.00;4.45]	2.80 [1.67;4.22]	0.394

*Chi-square test for categorical variables; Mann-Whitley test for numeric variables; Abbreviations: CAG: Community Adherence Group; HF: Health facility

**Fig 4:**
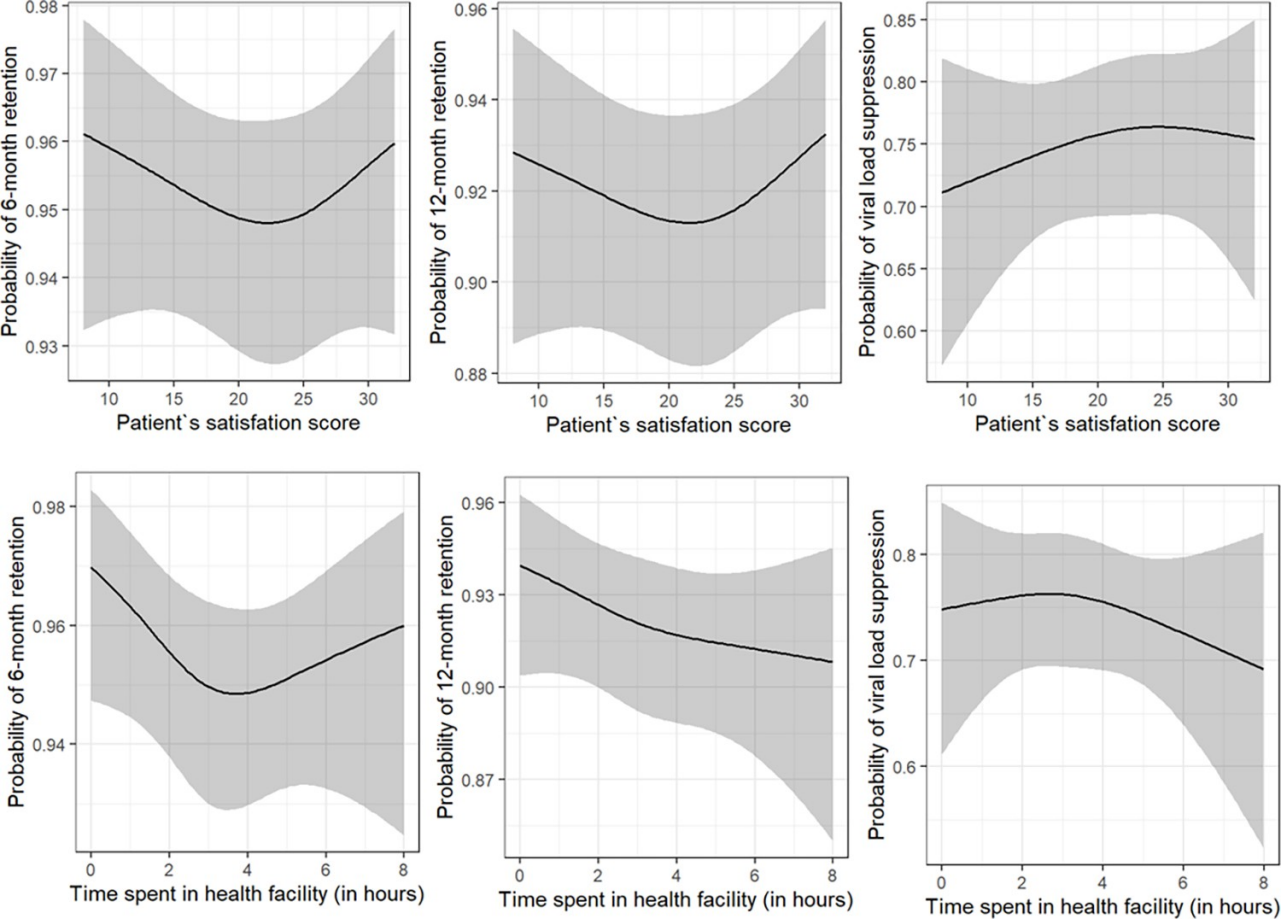
Effect of patient satisfaction score (A) and time spent in health facility (B) on the log-odds of being retained in care at 6 months (left), 12 months (middle) and the log-odds of viral suppression (right).

Results for the multivariable mixed-effects analysis are presented in **Table 4** and **Fig 4A** shows the non-linear effect of patient’s satisfaction score on log-odds of being retained at 6- and 12-months post-survey. As in the univariable case, patient satisfaction score was not associated with retention: when comparing the patient satisfaction score of 25 to 15, the odds of being retained at 6 months was 0.91 (95%CI: 0.69–1.20) and at 12 months was 0.97 (95%CI: 0.77–1.21). A similar approach was used to describe the (non-linear) effect of age: older patients had higher odds of being retained: the odds of 50-year-old patients being retained at 6- and 12-months were 59% and 28% higher compared to 30-year-old patients (Odds Ratio [OR] 1.59, 95%CI: 1.09–2.32 and OR 1.28, 95%CI: 0.96–1.70, respectively) (**Table 4**). We also performed a complete case analysis, excluding the variable marital status from our regression models. Results were similar and are presented in **S1 Table**.

**Table 4:** Multivariable mixed effects logistic regression to assess the impact of patient satisfaction on 6- and 12-month retention, and viral load suppression.

	6-month retention (n = 3,318)	p-value	12-month retention (n = 3,318)	p-value	Viral suppression (n = 1,718)	p-value
	OR (95%CI)		OR (95%CI)		OR (95%CI)	
**Satisfaction score**†		0.672^ꞎ^		0.947^ꞎ^		0.682^ꞎ^
10	1.15 (0.89; 1.47)		1.10 (0.89; 1.36)		0.85 (0.49; 1.46)	
15	Ref		Ref		Ref	
20	0.90 (0.73; 1.11)		0.93 (0.78; 1.11)		1.11 (0.83; 1.49)	
25	0.91 (0.69; 1.20)		0.97 (0.77; 1.21)		1.13 (0.82; 1.56)	
**Age at interview**		**0.002** ^ꞎ^		**<0.001** ^ꞎ^		**0.042** ^ꞎ^
20 years	0.73 (0.54; 0.99)		0.74 (0.58; 0.96)		0.93 (0.79; 1.10)	
30 years	Ref		Ref		Ref	
40 years	1.28 (1.11; 1.48)		1.18 (1.05; 1.32)		0.99 (0.79; 1.25)	
50 years	1.59 (1.09; 2.32)		1.28 (0.96; 1.70)		1.20 (0.89; 1.61)	
** Time on ART (years)**	1.27 (1.18; 1.38)	**<0.001**	1.28 (1.20; 1.36)	**<0.001**	0.94 (0.89; 1.00)	**0.027**
**Sex:** Male	0.57 (0.43; 0.74)	**<0.001**	0.97 (0.68; 1.38)	0.867	0.50 (0.35; 0.72)	**<0.001**
**Urban:** Yes	1.07 (0.81; 1.42)	0.642	1.02 (0.81; 1.29)	0.866	2.21 (1.53; 3.20)	**<0.001**
**Education:** Secondary or higher	0.78 (0.59; 1.04)	0.091	0.83 (0.65; 1.06)	0.135	0.95 (0.68; 1.33)	0.764
**Civil status:** Cohabitating	0.73 (0.54; 1.00)	**0.050**	0.71 (0.53; 0.93)	**0.022**	0.99 (0.71; 1.37)	0.953

†Satisfaction scores out of a possible total of 32. ^ꞎ^ P-value obtained from a likelihood-ratio test. Abbreviations: OR: odds ratio; CI: Confidence Interval; Ref: reference level; ART–antiretroviral treatment. Note: non-linear variables (satisfaction score and age at interview) were modelled using restricted cubic splines; inference was made via contrasts, following Shepherd et. al. [19], with reference values indicated in the table.

**Fig 5:**
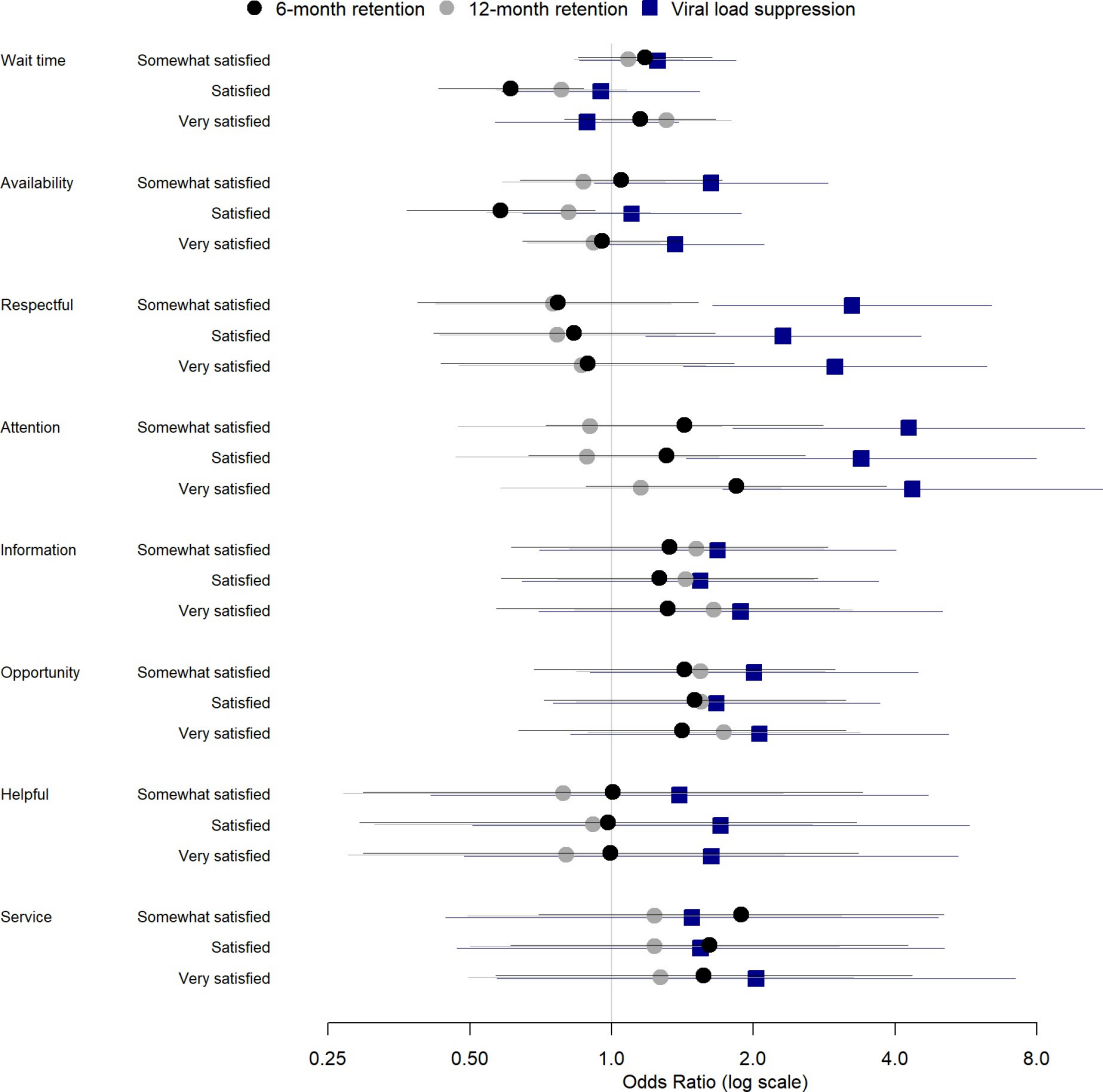
Effect of patient satisfaction score on viral suppression, 6- and 12-month retention; adjusted regression analysis.

The odds of being retained at 6- and 12-months were also not correlated with a higher patient satisfaction score regarding the individual questions (**Fig 5**).

### Satisfaction score and viral load

A total of 1,718 patients had viral load results within one year after taking the survey. The majority were women (74%), the median age was 27 years old (IQR 21–34), and the median satisfaction score across this sub-population was equal to 24 (IQR 16–26). There were 1,297 (75%) virally suppressed patients. They were, on average, older, female, and with a higher overall satisfaction score than the group of patients with a viral load above 1,000 counts/ml (*i*.*e*., not virally suppressed). Viral load was associated with the combined patient satisfaction score in a univariable analysis (**Table 3**), but not in a mixed-effects multivariable regression (OR 1.13, 95%CI: 0.82–1.56, for comparing 1 and 3 hours spent in HF) (**Table 4 and Fig 4A**); HF location seemed to have a large impact on the odds of being virally suppressed, with patients from urban areas having higher odds of being virally suppressed (OR 2.21, 95%CI: 1.53–3.20). We also noticed significant associations between patient satisfaction scores and the odds of being virally suppressed when analyzing individual questions separately. The forest plot in **Fig 5** shows that the provider interaction with the patient, mostly via respectfulness and attention, were strongly associated with higher odds of being virally suppressed. Analyses using a threshold of 50 copies/ml showed similar results (**S2 Table**).

#### Effect of time spent at the HF on patient satisfaction, retention to care and viral suppression

**Fig 6:**
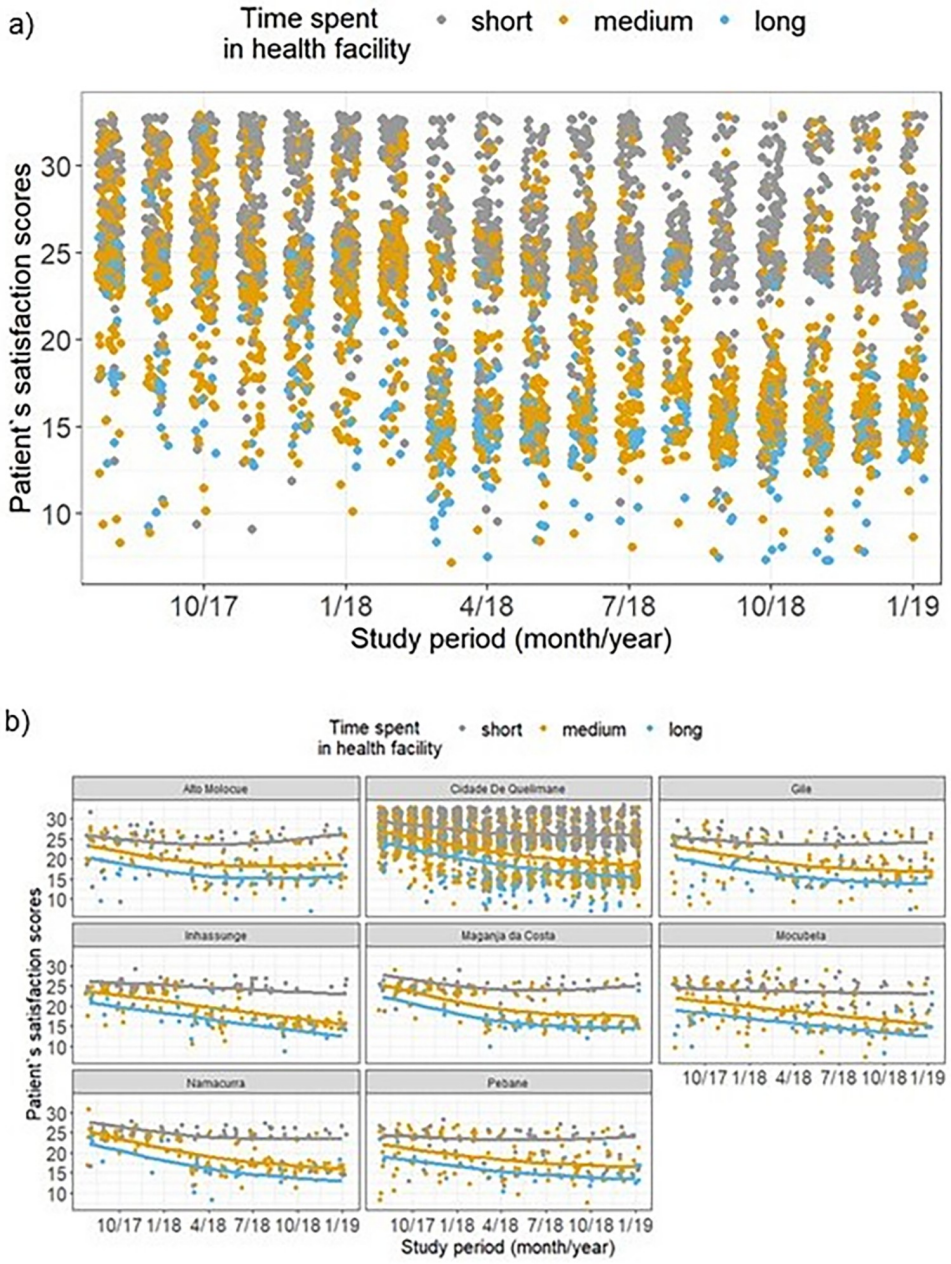
Patient satisfaction scores by time spent in health facility, overall (a) and per district (b). Lines correspond to the predicted values obtained from the ordinal regression, in which study period was modelled via restricted cubic splines, with three knots place at the 5^th^, 50^th^, and 95^th^ percentiles. Time spent: short (< 2.5 hours), medium (between 2.5 and 5 hours), and long (> 5 hours).

**Fig 6** suggests a separation of scores higher and lower than 20 between those who spent a short, medium or prolonged (long) time at the health facility. Patients that spent a short period of time in the HF (less than 2.5 hours) are clustered together at higher scores (above 20), while patients who experienced longer times (above 5 hours) are clustered together at low satisfaction scores. In other words, time spent in the HF was inversely correlated with patient satisfaction (Spearman’s correlation = -0.63, 95%CI: -0.61;-0.65), with similar values after stratifying by location of HFs: Spearman’s correlation = -0.57 (95%CI: -0.54;-0.60) and -0.53 (95%CI: -0.48;-0.57) for urban and rural sites, respectively. Varying the time spent in HF from 1 hour to 3 hours was somewhat related to a decrease in the overall satisfaction score of 17% (95%CI: 16%-18%). Results were very similar among men and women: Spearman’s correlation = -0.66 and -0.63, respectively, with a decrease in the satisfaction score of 17% (95%CI: 15% - 19%) among men and 17% (95%CI: 16% - 18%) among women with same variation of time at HF. This effect was sustained over time and was seen in all supported districts (**Fig 6**).

The impact of the time spent in HF was also evaluated on retention and viral suppression (**Fig 4B**). Varying the time spent in the HF was not associated with 6- or 12-month retention or viral suppression. Specifically, when varying the time spent in HF from 1 to 3 hours, there was a decrease of 28% (OR = 0.72; 95%CI: 0.52–1.01) and 17% (OR = 0.83; 95%CI = 0.63–1.09) in the odds of being retained in care at 6- and 12-months after the survey, with both 95% CI’s crossing 1.0. There was also no effect on viral suppression (OR = 1.02; 95%CI: 0.73–1.42). Please refer to **S3 Table** for details.

## Discussion

The main purpose of this study was to evaluate association between patient satisfaction and retention in HIV care. Overall, the participants gave high satisfaction scores for the services received at the select public health facilities, with some variation between the districts. Our findings are consistent with the results of patient satisfaction in Manica Province, another rural province in central Mozambique [23], and high scores have also been seen in other countries among PLWH [24–26]. This study was unique in the sense of having data over a period of 18 months, which enabled us to detect and show changes over time. A clear decline in satisfaction was seen after more and intense HF monitoring visits were done by health authorities and implementing partners (as of February 2018), potentially causing greater disruption in care for patients seeking services during those visits.

We did not see a significant association between patient satisfaction and retention in care at 6-, 12-months or viral suppression. A study in five SSA countries showed a marginal association between patient satisfaction and adherence, and no association with viral suppression [27].

Providers must take the time to adequately explain to patients the need for high levels of medication adherence and the importance of viral suppression in an effort to support patients’ long-term retention in HIV care and treatment. In Vietnam, responsiveness of health care workers to questions, and overall perceived satisfaction were related to an improvement of immunological status [24]. Wachira et al. described the importance of good provider-patient communication skills to improve patient satisfaction among unsuppressed patients [25]. Unfortunately, in SSA, patient-provider interaction is often characterized by poor communication, resulting in patient frustration and treatment abandonment [28].

As in other studies, we found that time spent in the HF (including wait time until being attended) strongly influenced patient satisfaction [27, 29]. Long wait times have consistently been reported as a barrier to and/or a common complaint among persons receiving HF-based care [23, 30, 31]. A pilot evaluation testing the option for scheduled (*i*.*e*., time-specific) health consultations in Mozambique showed acceptability and promise in reducing waiting times at HF [32]. Other differentiated models of care such as 3- or 6-monthly drug dispensation, community ART dispensation, 6-monthly clinical consultations, designed to be more patient-centered and provide greater options to patients have been expanded exponentially in recent years, may lead to an improved patient satisfaction with HIV services.

Other research has found positive patient satisfaction results related to the availability of laboratory [33], family planning [34], mHealth/SMS [35], nutrition [36], and extended-hours services for patients [37], as well as patient-centered companion policies [38]. Having patient feedback regarding specific services/interventions that are available, and assessing for potential associations with retention, could promote effective tailoring within programs.

The study had several limitations. The evaluation took place in a non-random selection of HF located in select districts within a single province, therefore the results may not be generalizable. Only patients enrolled in and attending health care services participated in the study, thus, those who may have already dropped out of care (potentially due to low levels of patient satisfaction) would not have been included in this survey-based analysis. There may have been measurement bias related to “time spent in the HF” as some patients arrived very early at their respective HF due to transport issues, and not necessarily related to delays being seen by the healthcare provider at the HF. Finally, the study was conducted in a period before differentiated models of care were expanded in Mozambique, and the association between patient satisfaction and retention could be different now with more patient-centered service delivery options.

## Conclusions

Overall, patient satisfaction was relatively high. While satisfaction was not associated with higher chance of retention to care and viral suppression, we saw that individual scores related to client-provider interaction regarding respect and attention to patients were associated with viral suppression. Additionally, patient satisfaction was driven largely by time spent at the health facility, which however was not associated with retention in care. Differentiated models of care to decongest crowded health facilities have been taken up to scale, however, continuous monitoring of patient satisfaction, retention and adherence to ART remains essential.

## Supporting information

S1 Fig Convergence diagnostics. (TIF)

S2 Fig Median patient satisfaction scores, by district and by month. (TIF)

S1 Table Complete case analysis, excluding the variable marital status from our regression models. (DOCX)

S2 Table Effect of satisfaction score on viral load, using a threshold of 50 copies/ml. (DOCX)

S3 Table Effect of time spent at health facility on retention and viral suppression. (DOCX)

S1 Dataset Dataset of patient survey. (CSV)

S1 Checklist STROBE statement—checklist of items that should be included in reports of observational studies. (DOCX)
