# Supplementary figures and images for: Time spent at health facility is a key driver of patient satisfaction, but did not influence retention to HIV care: A serial cross-sectional study in Mozambique
Source: PLoS One. 2024 Apr 18;19(4):e0299282. doi: 10.1371/journal.pone.0299282 (PMC11025808; doi:10.1371/journal.pone.0299282)

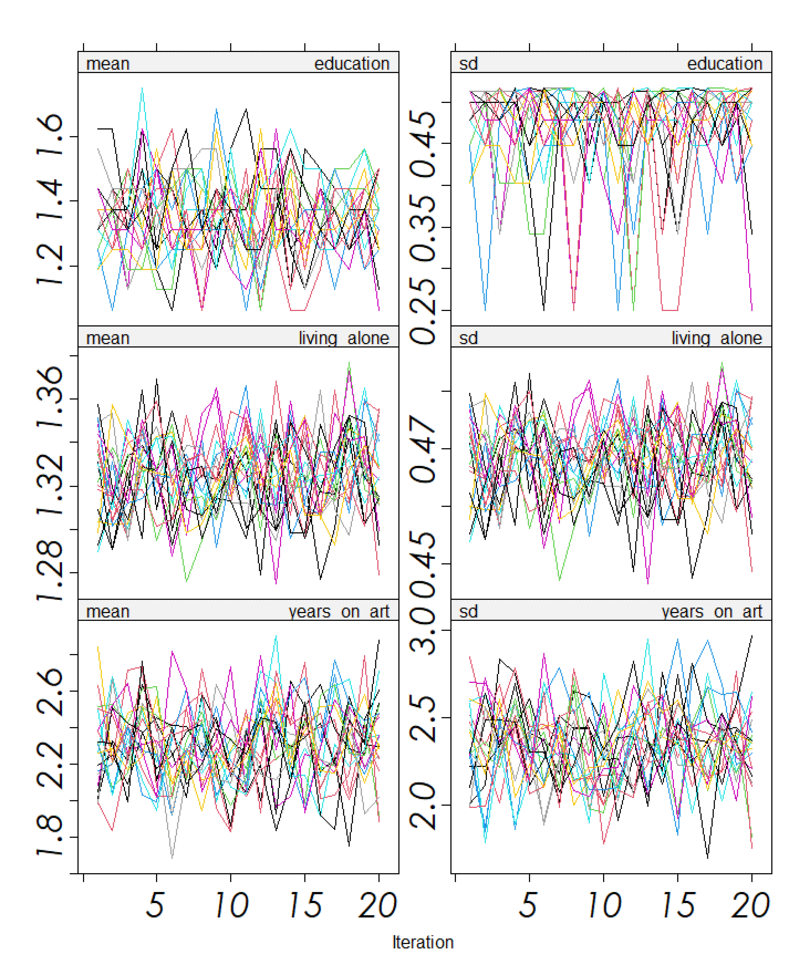

Supplement: S1 Fig — (TIF) [file pone.0299282.s001.tif]

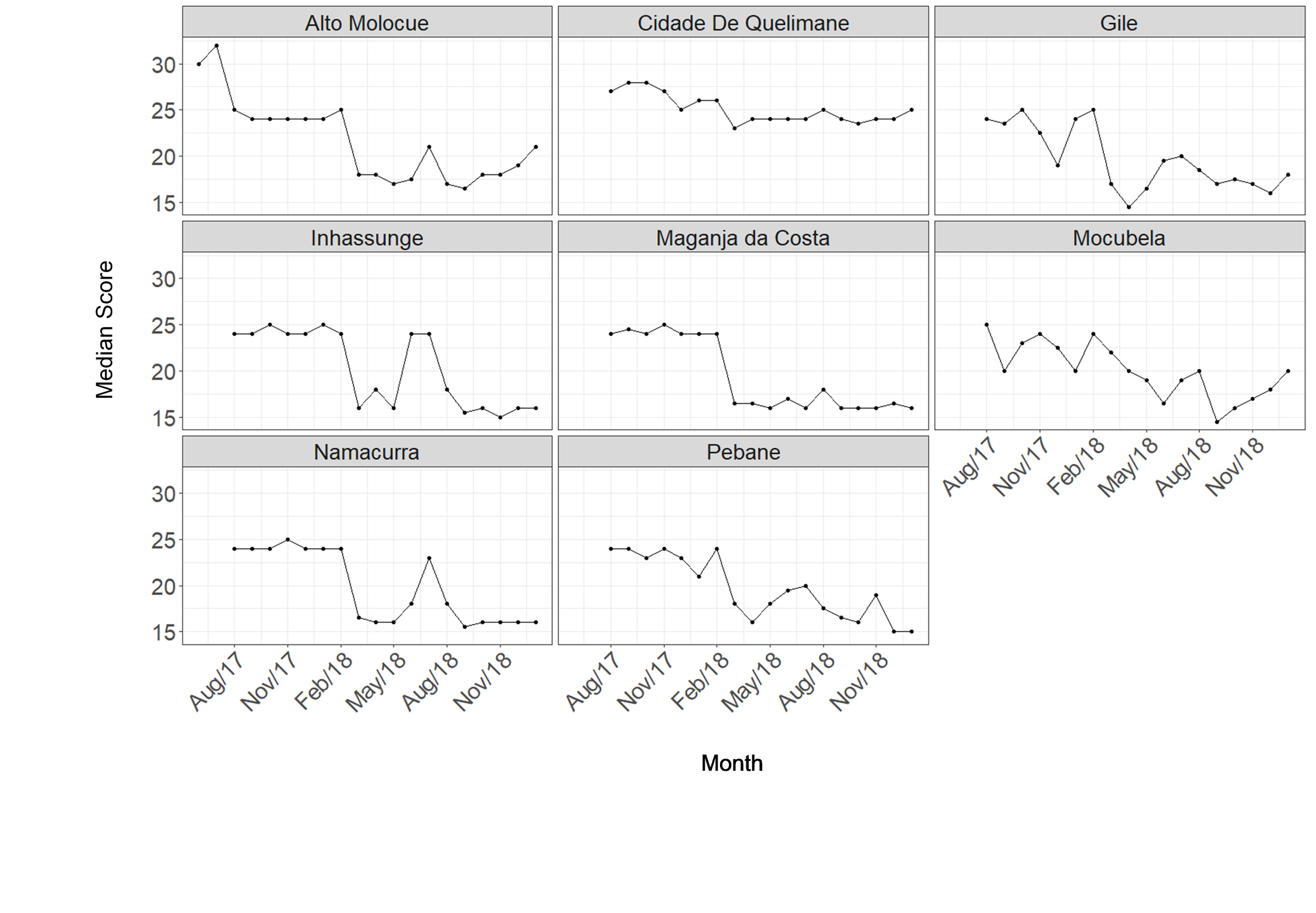

Supplement: S2 Fig — (TIF) [file pone.0299282.s002.tif]
