# Supplementary material for: Time spent at health facility is a key driver of patient satisfaction, but did not influence retention to HIV care: A serial cross-sectional study in Mozambique
Source: PLoS One. 2024 Apr 18;19(4):e0299282. doi: 10.1371/journal.pone.0299282 (PMC11025808; doi:10.1371/journal.pone.0299282)
Supplement: S1 Table — (DOCX) [file pone.0299282.s003.docx]

|  | **6-month retention**  **(n=3,318)** | **p-value** | **12-month retention**  **(n=3,318)** | **p-value** | **Viral suppression**  **(n=1,718)** | **p-value** |
| --- | --- | --- | --- | --- | --- | --- |
|  | **OR (95%CI)** |  | **OR (95%CI)** |  | **OR (95%CI)** |  |
| **Satisfaction score**† |  | 0.629 |  | 0.890 |  | 0.610 |
| 10 | 1.16 (0.90; 1.49) |  | 1.11 (0.90; 1.37) |  | 0.93 (0.73; 1.18) |  |
| 15 | Ref |  | Ref |  | Ref |  |
| 20 | 0.89 (0.72; 1.10) |  | 0.93 (0.78; 1.10) |  | 1.07 (0.87; 1.30) |  |
| 25 | 0.91 (0.69; 1.20) |  | 0.96 (0.77; 1.21) |  | 1.11 (0.86; 1.43) |  |
| **Age at interview** |  | **0.001** |  | **<0.005** |  | **<0.001** |
| 20 years | 0.73 (0.54; 0.99) |  | 0.74 (0.58; 0.96) |  | 0.53 (0.50; 0.62) |  |
| 30 years | Ref |  | Ref |  | Ref |  |
| 40 years | 1.28 (1.11; 1.48) |  | 1.17 (1.05; 1.31) |  | 1.13 (0.98; 1.30) |  |
| 50 years | 1.57 (1.08; 2.28) |  | 1.26 (0.95; 1.67) |  | 1.06 (0.76 1.47) |  |
| **Time on ART (years)** | 1.27 (1.18-1.38) | **<0.001** | 1.28 (1.20-1.36) | **<0.001** | 0.94 (0.89; 1.00) | **0.027** |
| **Sex:** Male | 0.57 (0.44-0.75) | **<0.001** | 0.61 (0.49-0.77) | **<0.001** | 0.50 (0.35; 0.72) | **<0.001** |
| **Urban:** Yes | 0.89 (0.64-1.25) | 0.489 | 0.94 (0.66-1.33) | 0.732 | 2.21 (1.53; 3.20) | **<0.001** |
| **Education:** Secondary or higher | 0.77 (0.58-1.02) | 0.071 | 0.81 (0.64-1.03) | 0.080 | 0.99 (0.71; 1.37) | 0.953 |

**Supplementary Information S3.** Multivariable logistic regression to assess the impact of patient satisfaction on 6- and 12-month retention, and viral load suppression, without adjusting for marital status.

†*Satisfaction scores out of a possible total of 32. Abbreviations: OR: odds ratio; CI: Confidence Interval; Ref: reference level; ART: antiretroviral therapy*
