# Supplementary material for: Time spent at health facility is a key driver of patient satisfaction, but did not influence retention to HIV care: A serial cross-sectional study in Mozambique
Source: PLoS One. 2024 Apr 18;19(4):e0299282. doi: 10.1371/journal.pone.0299282 (PMC11025808; doi:10.1371/journal.pone.0299282)
Supplement: S2 Table — (DOCX) [file pone.0299282.s004.docx]

**Supplementary Information S4.** Multivariable logistic regression to assess the impact of patient satisfaction on viral load suppression, using threshold of 50 copies/ml.

|  | **Viral suppression** | **p-value** |
| --- | --- | --- |
|  | **(n=1,718)** |  |
|  | **OR (95%CI)** |  |
| **Satisfaction score**† |  |  |
| 10 | 0.95 (0.58-1.57) | 0.959 |
| 15 | Ref |  |
| 20 | 1.02 (0.78-1.33) |  |
| 25 | 1.00 (0.74-1.34) |  |
| **Age at interview** |  | 0.026 |
| 20 years | 0.95 (0.88-1.02) |  |
| 30 years | Ref |  |
| 40 years | 0.99 (0.89-1.09) |  |
| 50 years | 1.15 (1.00-1.33) |  |
| **Time on ART (years)** | 0.96 (0.91-1.01) | 0.134 |
| **Sex:** Male | 0.60 (0.43-0.83) | 0.003 |
| **Urban:** Yes | 2.33 (1.69-3.22) | <0.001 |
| **Education:** Secondary or higher | 0.95 (0.70-1.29) | 0.742 |
| **Civil status:** Living alone | 1.03 (0.76-1.39) | 0.845 |

†*Satisfaction scores out of a possible total of 32. Abbreviations: OR: odds ratio; CI: Confidence Interval; Ref: reference level; ART: antiretroviral therapy*
