# Supplementary material for: Time spent at health facility is a key driver of patient satisfaction, but did not influence retention to HIV care: A serial cross-sectional study in Mozambique
Source: PLoS One. 2024 Apr 18;19(4):e0299282. doi: 10.1371/journal.pone.0299282 (PMC11025808; doi:10.1371/journal.pone.0299282)
Supplement: S3 Table — (DOCX) [file pone.0299282.s005.docx]

**Supplementary Information S5.** Multivariable logistic regression to assess the impact of time spent in health facility on 6-months retention, 12-months retention, and viral load suppression.

|  | **6-month retention**  **(n=3,318)**  **OR (95%CI)** | **12-month retention**  **(n=3,318)**  **OR (95%CI)** | **Viral suppression**  **(n=1,718)**  **OR (95%CI)** |
| --- | --- | --- | --- |
| **Time spent in health facility (hours)** |  |  |  |
| 1 | Ref | Ref | Ref |
| 2 | 0.82 (0.67-1.01) | 0.90 (0.77-1.07) | 1.04 (0.81-1.33) |
| 3 | 0.72 (0.52-1.01) | 0.83 (0.63-1.09) | 1.02 (0.73-1.42) |
| 4 | 0.71 (0.50-1.01) | 0.79 (0.59-1.05) | 1.06 (0.60-1.88) |

*Abbreviations: OR: odds ratio; CI: Confidence Interval; Ref: reference level.*
